# MEA Viewer: A high-performance interactive application for visualizing electrophysiological data

**DOI:** 10.1371/journal.pone.0192477

**Published:** 2018-02-09

**Authors:** Daniel C. Bridges, Kenneth R. Tovar, Bian Wu, Paul K. Hansma, Kenneth S. Kosik

**Affiliations:** 1 Neuroscience Research Institute, University of California at Santa Barbara, Santa Barbara, California, United States of America; 2 Department of Physics, University of California at Santa Barbara, Santa Barbara, California, United States of America; Georgia State University, UNITED STATES

## Abstract

Action potentials can be recorded extracellularly from hundreds of neurons simultaneously with multi-electrode arrays. These can typically have as many as 120 or more electrodes. The brief duration of action potentials requires a high sampling frequency to reliably capture each waveform. The resulting raw data files are therefore large and difficult to visualize with traditional plotting tools. Common approaches to deal with the difficulties of data display, such as extracting spike times and performing spike train analysis, are useful in many contexts but they also significantly reduce data dimensionality. The use of tools which minimize data processing enable the development of heuristic perspective of experimental results. Here we introduce MEA Viewer, a high-performance open source application for the direct visualization of multi-channel electrophysiological data. MEA Viewer includes several high-performance visualizations, including an easily navigable overview of recorded extracellular action potentials from all data channels overlaid with spike timestamp data and an interactive raster plot. MEA Viewer can also display the two dimensional extent of action potential propagation in single neurons by signal averaging extracellular action potentials (eAPs) from single neurons detected on multiple electrodes. This view extracts and displays eAP timing information and eAP waveforms that are otherwise below the spike detection threshold. This entirely new method of using MEAs opens up novel research applications for medium density arrays. MEA Viewer is licensed under the General Public License version 3, GPLv3, and is available at http://github.com/dbridges/mea-tools.

## Introduction

Multi-electrode arrays (MEAs) can record extracellular action potentials (eAPs), also known as spikes, from neuronal ensembles *in vitro* and *in vivo*, and are increasingly used in research areas as diverse as neuronal connectivity and drug discovery [1]. Because the duration of transmembrane action potentials ranges from 0.2 to 4 milliseconds, depending on the neuron type, the sampling frequency required to reliably capture eAPs is commonly in the range of 10 to 25 kHz. Thus in arrays with 120 electrodes, experiments generate data at a rate of over 300 MB/min, resulting in large unwieldy files. Steps that simplify the task of data handling, such as extracting eAP timing information by spike detection and subsequent sorting, also dramatically reduce data dimensionality. Additionally, spike detection and sorting steps can be notoriously error prone [2] and information present in the lower frequency domains of analog voltage recordings is not captured by spike detection. Methods and tools that, for example, display superimposed spike-detected and sorted data on the extracellular voltage recording could be used to evaluate the performance of detection and sorting algorithms in an unbiased way and could help avoid systematic errors by quickly identifying detection and sorting failures. Such a data display could reveal features in the raw data that are otherwise difficult to access in processed data.

Here we introduce MEA Viewer, a software package for high-performance visualization of multi-channel electrophysiological data recorded with multi-electrode array systems. MEA Viewer was written to be a general-purpose electrophysiology data display application for large (0.5–1.5 GB) multi-stream data files. The primary audience for MEA Viewer is end users of MEA systems who want to explore their data prior to doing heavy statistical analysis. MEA Viewer fills the void for tools to easily visualize unprocessed data. MEA Viewer is ideal for examining the performance of spike sorting algorithms because it superimposes extracellular voltage data with the results of spike detection and spike sorting routines.

**Fig 1:**
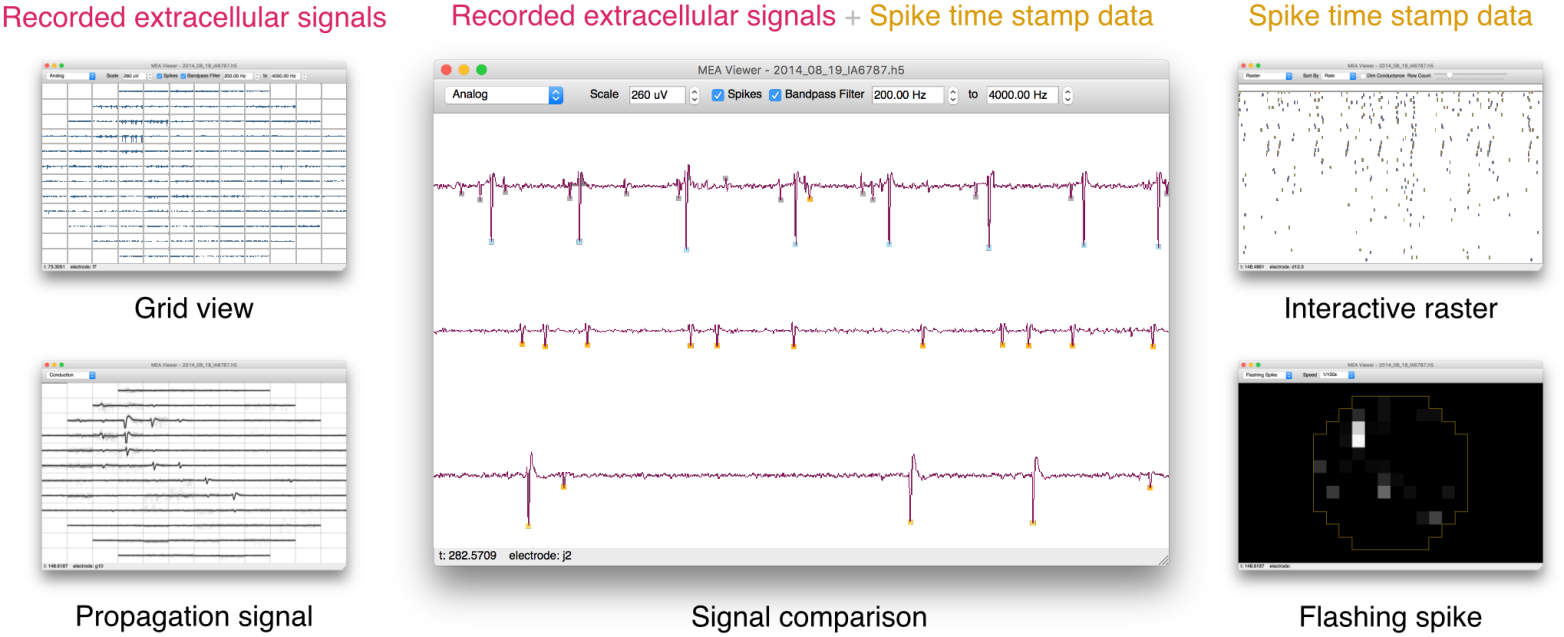
MEA Viewer provides 5 interactive visualizations of electrophysiological data. Recorded extracellular signals from 120 electrodes (top left) and spike timestamp data (top right) can be overlaid (middle) for unbiased investigation of MEA data. Multiple views are provided to easily navigate the entirety of the recorded signals, providing the ability to probe spike timing relations (bottom right), view and identify signals consistent with axonal propagation (bottom left), and verify spike detection and sorting performance (middle).

MEA Viewer provides five main visualization interfaces (Fig 1): (i) the grid view displays the complete set of recorded extracellular signals, (ii) the signal comparison displays multiple selected channels with superimposed spike data, (iii) an interactive raster view displays spike timestamp data for all recorded channels, (iv) the flashing spike view presents a spatial-temporal representation of the spiking behavior of the recorded channels at user-specified time windows, at a number of play-back speeds (v) the propagation signal view displays signal-averaged spikes from single neurons, revealing the two dimensional patterns of action potential propagation across the array, as well as eAPs otherwise typically masked by recording noise.

Assigning eAPs to a particular neuron is not typically possible when recording extracellularly. However, we recently showed that MEAs can be used to monitor action potential propagation in single neurons by the co-detection by multiple electrodes of eAPs in different parts of the same neuron [3]. Stereotyped co-detection confirms that these events arise from single neurons and their patterns in time and space uniquely identifies each neuron that underlies these signals. By revealing the presence of propagation signals, MEA Viewer can be used to unambiguously identify and explore the spiking behavior from single identified neurons, making MEA Viewer the first software of its kind to do so. MEA Viewer can use co-detected eAPs from nearby electrodes to extract and average repeated spiking events, revealing signals in channels previously masked by noise. Retaining these previously undetected events enables commonly used medium-density arrays (100–200 um electrode pitch) to detect action potential propagation from single axonal arbors in a high-throughput way. Because extracellular recording is non-invasive, the spiking of uniquely identified neurons can be followed over multiple days. This may be especially useful for experiments that monitor changes due to degeneration and in disease models.

## Design and implementation

The high performance of MEA Viewer is achieved by transferring a majority of the data processing to the graphics processing unit (GPU). MEA Viewer was written in Python 3 and OpenGL Shading Language, making extensive use of the Python scientific stack (numpy, scipy, pandas, and h5py). Most of the interactions with the GPU are done with vispy [4], a high-level Python/OpenGL interface to modern graphics hardware. The rest of the user interface was created using PyQt and the Qt GUI library, enabling the application to operate across platforms (currently tested on Windows 7 and Mac OS X 10.10+).

**Fig 2:**
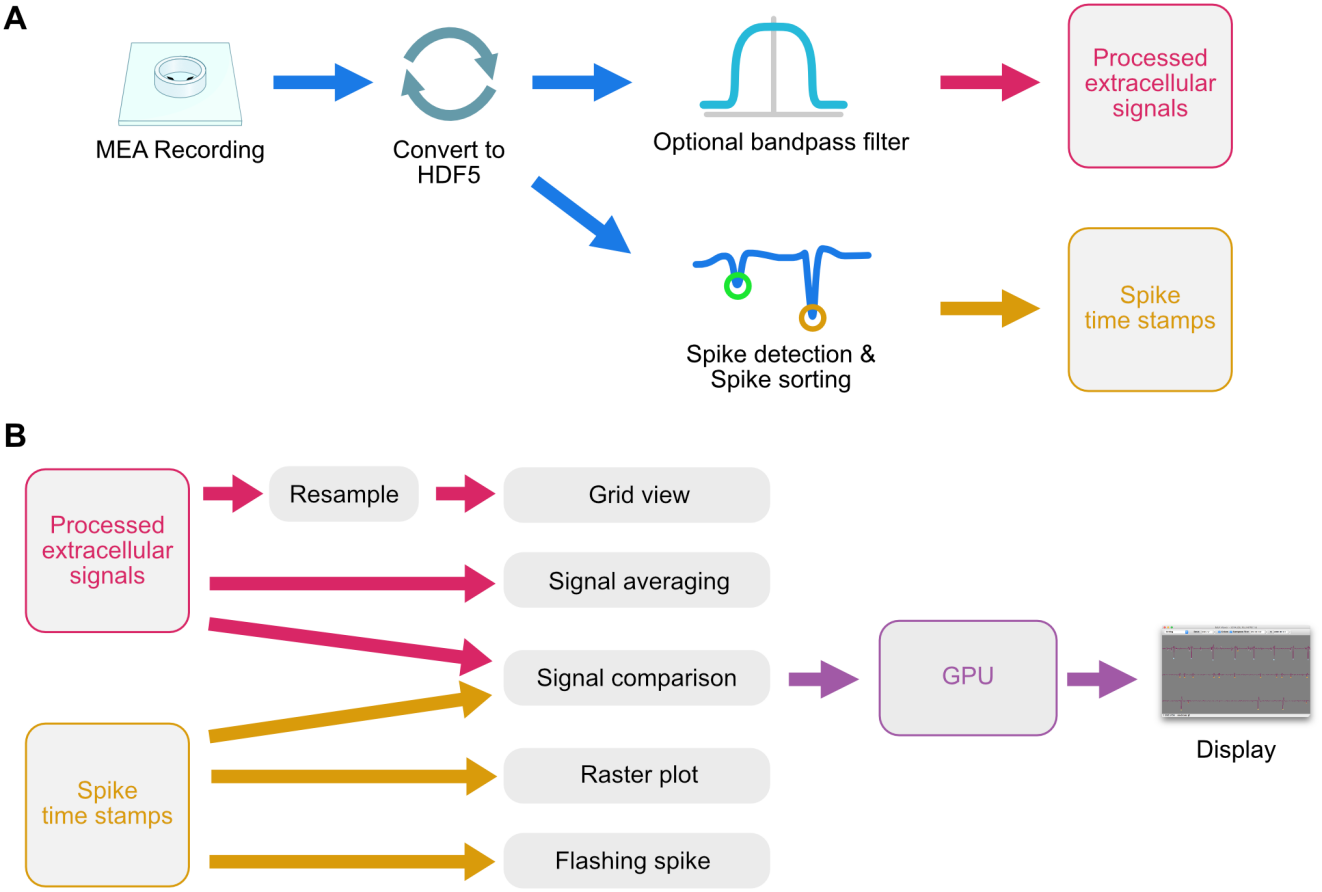
Pre-processing and display workflow used by MEA Viewer. (A) Extracellular signals are recorded with a commercial MEA system, then converted to a Hierarchical Data Format (HDF5). Data can then be optionally filtered and processed by a spike detection and spike sorting engine. These steps occur outside of MEA Viewer, but can be completed with tools in the overall mea-tools package. (B) The recorded extracellular signals and spike time stamp data are imported into MEA Viewer and loaded into memory. Due to the large number of data points, recorded signals are resampled prior to display in the grid view. Once a user selects their desired visualization, MEA Viewer generates vertex data that is sent to the GPU of the host computer, then displayed.

A brief overview of the data-conditioning steps required to use MEA Viewer is shown in Fig 2A. MEA Viewer accepts extracellular voltage data in the form of Hierarchical Data Format version 5 (HDF5) files and comma-separated value (CSV) files for spike timestamps. HDF5 is an open file format stewarded by the HDF Group, and is the file format adopted by the Neuroscience without Borders initiative [5]. We generate spike timestamp data using tools available with MEA Viewer. However, there is a vast number of options in spike sorting routines, with no clear sorting method or implementation widely adopted. Therefore, rather than forcing end-users to use our spike sorting protocols, we chose instead to utilize the common CSV file format to maximize interoperability for other users. Spike timestamp data can be generated using our tools, or from any other spike detection and sorting program, then converted to a CSV file compatible with MEA Viewer (see S1 File). MEA Viewer interoperates well with our data acquisition system (MCS MEA2100 for 120 electrodes), but with its use of open data formats has the ability to support a variety of systems.

Once input data is formatted for reading by MEA Viewer, GPU vertex data is created by the currently active visualization and sent to the GPU for rendering (Fig 2B). Most visualizations create vertex data for the entirety of the dataset displayed and transfer it once to a vertex buffer object on the GPU. To allow for panning and zooming a transformation matrix is then updated each animation frame, eliminating the massive transfer of vertex data on a frame-by-frame basis. One notable exception to this methodology is with the grid view which displays the entirety of the originally recorded extracellular signals. Because the grid view often displays overviews of a gigabyte or more of analog data, it is impractical to send all of this to the GPU at one time due to memory limits on consumer grade GPU cards. Instead the analog data is resampled and updated during mouse drag and scroll events to only transfer the selected data. Because the number of data points encompassing the record is much larger than the number of pixels used to display it, data is typically downsampled for display. When dealing with long-duration recordings of spiking neurons it is important to downsample in a way that preserves an accurate view of these relatively sparse events. Transferring every n^th^ point makes it unlikely for these points to land on spikes—creating a visualization that underestimates the actual spiking behavior. We sidestep this by using a simple method to downsample by calculating the number of pixels used to display each waveform (n_p_), then binning the waveform data into n_p_ bins. The minimum and maximum value for each bin are calculated and those points are sent to the GPU as vertex data, which is then displayed as an OpenGL line strip. This design preserves an accurate view of the spiking behavior and reduces the total amount of data being transferred to manageable levels, preserving a fluid interaction with the data.

The grid, propagation signal, and flashing spike visualizations display data in a layout that mimics the geometry of the MEA. Custom layouts can be specified in the software by subclassing the abstract Layout class and implementing the methods coordinates_for_electrode and electrode_for_coordinates, as well as providing rows and columns attributes specifying the number of rows and columns in the layout. These functions are mostly self explanatory, with all coordinate values given in (column, row) tuples and electrode values given as strings. This functionality allows electrophysiological data from any sized and shaped array to be visualized in a way that corresponds to the electrode’s physical location on the MEA.

## Results

**Fig 3:**
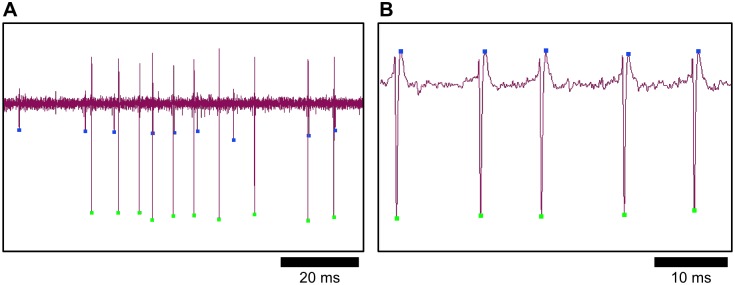
A signal comparison view displays recorded extracellular signals with overlaid spike timestamp data. (A) The signal comparison view allows for the inspection of spike detection and spike sorting accuracy. Here, two distinct waveforms are present and correctly sorted (see blue and green markers on small and large amplitude spikes, respectively). (B) An example showing a systematic error in spike detection on this large-amplitude signal. A false positive spike detection (blue) consistently occurs during the re-polarization phase of the action potential. Errors in spike processing like these are hard to detect without comparisons to the raw original recording and can have large effects on computed spike train statistics.

The entry point for displaying extracellular voltage data with MEA Viewer is the grid view (Fig 1). From this window, users can scan the recorded analog data from all electrodes, easily pan forward and reverse in the time record and zoom in and out at chosen locations in time and user-specified signal amplitudes. Users can select individual channels to inspect or a comparison view of user-chosen channels can be activated to display extracellular voltage overlaid with spike detection and sorting data (Fig 3A). Detected spikes are color-coded by markers indicating sorted spike groups. Spikes from single neurons that are detected on multiple electrodes are indicated by gray markers. MEA Viewer also enables the rapid visualization of redundant spike information, which can be underappreciated when analyzing MEA data [3]. The obvious utility of this visualization in MEA Viewer is that common spike-detection and -sorting routines can be easily assessed in this view (Fig 3B). Navigating through the data in this way allows for unbiased assessment of spike detection and sorting performance of any data record. As detection and sorting errors can have large effects on measured spike rates, MEA Viewer can be used to spot cases like these early in the data analysis process.

Raster plots are useful for displaying array-wide activity patterns. However, a typical raster plot displaying 3 minutes of data does so at a resolution of ~130 ms/px on a 1400 pixel monitor. These displays can thus significantly undersample the displayed data. Considering spiking can exceed 20 Hz during bursts, a sample window of 130 ms could contain 2–3 individual events, but would only display as a single event on the raster plot. MEA Viewer sidesteps the limitations of fixed-scale raster plots by displaying spike dynamics at user-chosen time scales. The dynamic raster view in MEA Viewer displays spike time data from all data channels or from user-chosen channels and can display spike-sorted or unsorted data. MEA Viewer also offers many channel-ordering schemes which define a channel’s row position to facilitate exploratory investigation. To provide a clearer view of array spiking dynamics, data in the interactive raster view can be ordered by activity level, with the most active channels on top, by their spike latency from an arbitrary time or by a fixed electrode order.

The flashing spike view displays the spatial-temporal patterns of spike activity for user-chosen time windows. Array activity is animated by flashing a marker at a channel’s physical position when a spike arrives at that channel. Playback time is adjustable (from real time to 1/1600 of real time), to easily observe the dynamics among active channels. As subsequent spikes arrive, the spatial and temporal characteristics present are obvious. This view displays the spatial and temporal characteristics of spiking in array-wide data, as well as demonstrating the very short inter-electrode spike latency and stereotypy characteristic of propagation signals in contrast to array-wide spiking (see S1 Movie). The flashing spike view is useful, for example, when the temporal spiking patterns are important to superimpose on spatial electrode profiles [6].

MEA Viewer’s user interface makes it easy to switch between any of the data visualizations while maintaining a consistent position in the data record. For instance, after panning to a specific part of the data in the interactive raster view, switching to the flashing spike view will show data from that same time point. This makes it easy to coherently navigate the data record and rapidly switch between views of the original extracellular recordings and views of the eAP timestamp data.

**Fig 4:**
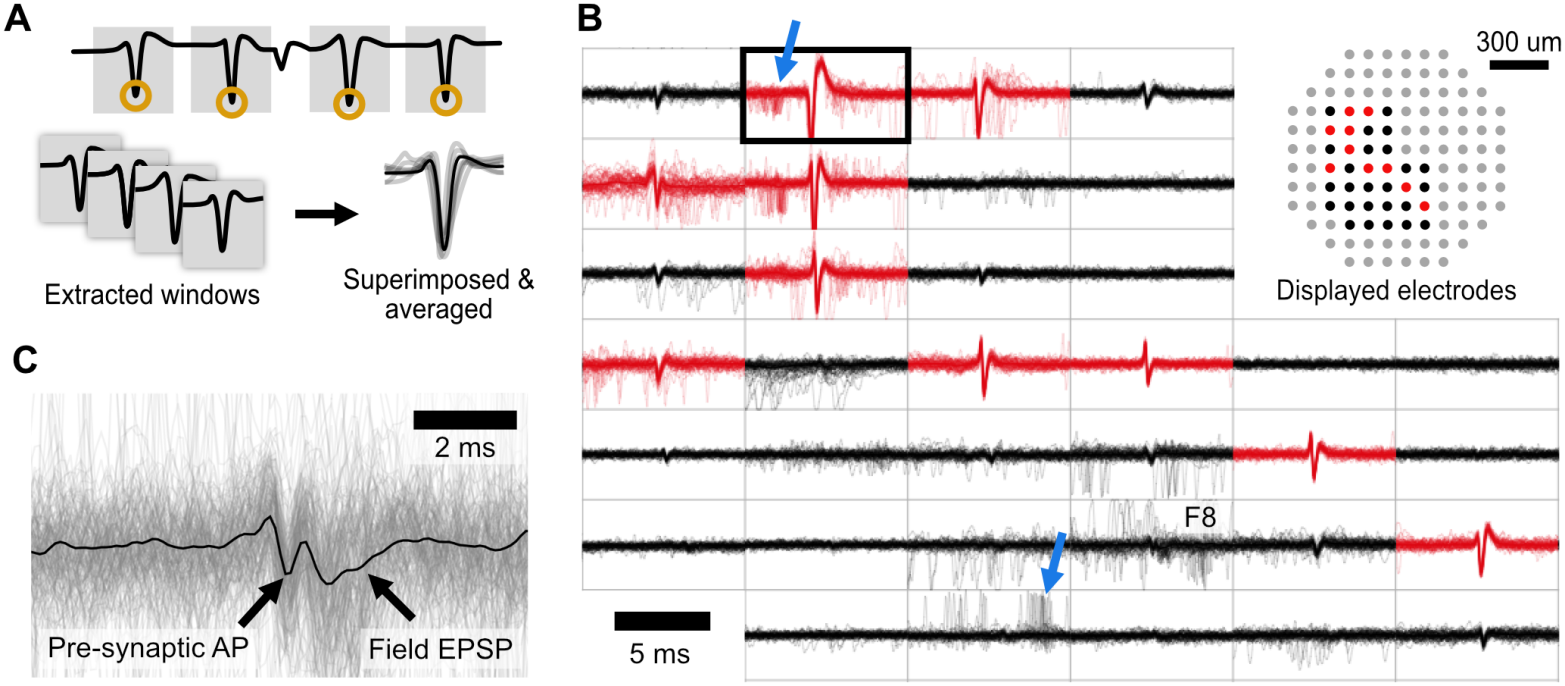
The propagation signal view overlays aggregated waveforms from all channels during repeated spike events from a reference channel, revealing axonal propagation and coupled pre- and post-synaptic spiking. (A) The reference channel (black outline) can be chosen as a single spike-sorted channel or two coincident channels. For each spike time in the reference channel, a 20 ms window is extracted from all other channels. The collected windowed waveforms for each channel are superimposed and averaged providing an overview of time-locked events across the array. (B) Example traces from cultured mouse hippocampal neurons grown on a 120 electrode count MEA (100 μm inter-electrode spacing), with the reference alignment channel outlined in bold. Channels shown in red have spike magnitudes above spike detection threshold. In this example, there are numerous channels exhibiting time-locked spiking with the reference channel (well-defined central spikes, in red). These events are due to action potential propagation down an axon, originating from the top left corner and propagating to the bottom right. A cloud of spikes preceding (top blue arrow) and following (bottom blue arrow) the propagation signal are also visible, consistent with expectations for presynaptic and postsynaptic partners of the reference neuron, respectively. Relationships such as these revealed by MEA Viewer can be subsequently confirmed experimentally. (C) Enlarged and rescaled view of electrode labeled F8 in (B). Signal averaging reclaims features typically masked by noise, in this instance a waveform consistent with a excitatory postsynaptic potential (EPSP) and possibly the presynaptic AP are revealed.

We recently showed that low density electrode arrays can be used to detect action potential propagation within single neurons in culture [3]. Propagation events are evident by the near-coincident and stereotyped spiking patterns among electrodes and we routinely recorded action potential propagation on multiple electrodes of arrays with inter-electrode distances of 100–200 um. A novel feature of MEA Viewer is that it easily displays cases where multiple electrodes detect action potential propagation. Because propagation signals arise from single neurons, the individual eAPs can be aligned and signal averaged. MEA Viewer signal averages single neuron eAPs by comparing event times of two channels when they show stereotyped coincident spiking (defined by repeated events within 0.7 ms of each other). The first channel is the ‘reference’ and the second is the ‘test’ channel. The reference channel can alternatively be chosen by extracting spike times from single sorted channels. Once a list of spike times is specified, a 20 ms window of data is extracted from every data channel, centered on each spike time in the reference channel (Fig 4A). The resulting ensemble of channels with highly correlated eAPs reflect action potential propagation within a single neuron measured at different locations across the entire array. The propagation signal view in MEA Viewer overlays aggregated spike waveforms to visualize propagation in single neurons across arrays, along with other associated events occurring close in time to spikes in identified cells. In this example, the earliest action potential is in the top left and propagates to the bottom right (Fig 4B). Averaged waveforms for each data channel are superimposed with individual waveforms, and waveforms from all data channels are displayed in a layout mirroring the MEA geometry. Signal averaging hundreds of spikes in this way reveals electrodes with sub-threshold eAPs and gives an overview of behavior at all electrodes time-locked to the spike events occurring in the reference channel (Fig 4C). Thus, non-invasive extracellular recording can be used to extract single neuron spiking data by the isolation of propagation signals.

The propagation signal view reveals other network events that are time-locked to spiking in the propagation signal. An example of this is the presence of spikes from other units that occur with timing expected from neurons with direct pre- or postsynaptic coupling to the neuron giving rise to the propagation signal (Fig 4B). Such events are distinguished from propagation signals by their longer time delay (between 1 and 10 ms) and their increased jitter with respect to the reference electrode, shown by the cloud of spikes seen in the overlaid waveforms (Fig 4B, blue arrows). In contrast, eAPs in different regions of the single neurons measured by multiple electrodes have relatively little jitter, and superimpose well over multiple instances [3]. Signal averaging may also unmask features typically obscured by noise, for instance a signal consistent with the characteristics expected from excitatory post-synaptic potentials (EPSP; Fig 4C), something normally not detected by extracellular recording of cultured neurons.

## Discussion

MEA Viewer was written to give users an interactive path for exploratory analysis of multi-stream data files. MEA Viewer is meant for the rapid initial investigation of recorded electrophysiological data and gives users the ability to display the dynamics of the system under study in preparation for subsequent statistical analysis. MEA Viewer differs from software packages such as the MATLAB toolbox MEA-Tools [7] or the commercial NeuroExplorer which focus heavily on statistical analysis of spike timestamp data without providing views of the analog data from all electrodes simultaneously. The open-source visualization tool, NeuroScope [8], displays continuous time signals, but does not offer the performance of MEA Viewer to quickly navigate large data sets. Software provided by MultiChannel Systems replays recorded data from all channels in a single playback direction and does not have the ability to easily compare user-chosen channels, or to zoom and pan through the dataset. MEA Viewer is unique in that no other applications support visualization of signal-averaged propagation signals. These combined features facilitate novel investigations of MEA data.

MEA Viewer is an open source application (licensed under the GPLv3), and was written to support open data formats. Spike data is read from plain text CSV files, and the HDF5 format was chosen for storing analog data due to its open specification. Other projects such as NEO [9], G-Node [10], or NSDF [11] provide libraries to read electrophysiological data from a variety of formats, but without there being a clear standard among the three we chose to focus our efforts on supporting HDF5 files. Both of these format specifications are fully detailed in S1 File.

The data visualizations in MEA Viewer present array-wide spike dynamics in an unprocessed and unbiased way, and permit other less spike timing-centric analyses. Exploratory data visualization can identify errors early on in analysis workflow, and is therefore vital before subsequent processing. A variety of complementary data visualizations enhances the user’s intuition of the system under study and allows for rapid hypothesis generation and more robust testing. MEA Viewer provides an attractive entry point for examining the topology of features present in extracellular recordings, from traditional spike train analysis to observation and identification of single neuron propagation signals.

The ability to examine the biophysics of action potential propagation with the propagation signal view creates novel opportunities for investigating diseases or compounds that affect axonal propagation or ion channel distribution and enables low density MEAs to provide novel data in addition to traditional spike train analysis. For example, experiments designed to examine the developmental progression of axon excitability, in neurons derived from different iPSC lines, are possible. Measurement of action potential propagation with multi-electrode arrays has been previously reported, but with high-density arrays composed of thousands of electrodes [12], or by more technically challenging experiments using dual patch electrodes [13]. We have shown that low density arrays can also be used to assess propagation [3]. Once MEA Viewer identifies propagation signals, subsequent analysis (e.g., developmental changes of electrophysiological properties), can occur in any analysis software of choice.

Because of its general applicability MEA Viewer is an attractive platform on which to build additional features. In its current state, MEA Viewer is primarily intended for exploratory data visualization whereby the large data files produced by MEA recordings can be easily examined prior to subsequent statistical approaches. Future visualizations in MEA Viewer will focus on displaying data interpretation alongside the currently implemented views.

The application of network modeling techniques to spike train data is one area that could benefit substantially from a simplified user interface, enabling more widespread adoption of these sophisticated techniques. Dynamic modeling approaches which produce functional networks, like the kinetic Ising model and generalized linear model [14–16], are powerful techniques for spike train analysis, but are not easily accessible. MEA Viewer could be extended to provide an intuitive interface to refine input parameters of various network models, and display an attractive view of the resulting network. Users would then be able to perturb the generated functional model—for example, by removing individual neurons, groups of nodes, or specific connections within the network—and see the resulting output in the network display. Additional features such as these would only improve the usefulness of the already implemented core functionality of MEA Viewer.

## Methods

### Cell culture

All animal procedures were approved by the University of California’s institutional IACUC protocol, in accordance with NIH policy. Neurons were prepared from C57BL/6 mice. Briefly, postnatal day 1 mice were decapitated, hippocampi were dissected, dissociated using papain for 30 minutes followed by trituration, and plated at a density of 550 cells/mm^2^ on a confluent bed of primary mouse glia. Cells were incubated at 37 °C with 5% CO_2_ and maintained in Minimum Essential Medium supplemented with 5% heat-inactivated fetal calf serum and Mito+ serum extender.

### MEA recordings

Extracellular recordings were obtained using a Multi Channel Systems (MCS, Reutlingen, Germany) MEA-2100 system and a 120-count multi-electrode array with 100 μm inter-electrode spacing and 30 μm electrode diameter, at a sampling frequency of 20 kHz.

### Spike detection/spike sorting

MultiChannel Systems proprietary files were converted to HDF5 file format prior to all analysis. Extracellular signals were bandpass filtered using a digital 2nd order Butterworth filter with cutoff frequencies of 0.2 and 4 kHz. Spikes were then detected using a threshold of 6 times the standard deviation of the median noise level. Spike sorting was performed by extracting 3ms long waveforms from each detected spike location, reducing dimensionality with PCA, and finally clustering using the OPTICS algorithm.

## Supporting information

S1 File Data format specifications. Specifications for spike and analog data formats accepted by MEA Viewer.
(DOCX)

S1 Movie Flashing spike visualization. Detected spikes are visualized as flashes at each electrode and played back at reduced speed to visualize the dynamics of the network. In this color enhanced movie the dynamics of 3 neurons are clearly seen, including their axonal propagation signals and likely synaptic coupling.
(MOV)
