# Supplementary material for: MEA Viewer: A high-performance interactive application for visualizing electrophysiological data
Source: PLoS One. 2018 Feb 9;13(2):e0192477. doi: 10.1371/journal.pone.0192477 (PMC5806868; doi:10.1371/journal.pone.0192477)
Supplement: S1 File — Specifications for spike and analog data formats accepted by MEA Viewer. (DOCX) [file pone.0192477.s001.docx]

**Supplementary Material**

*Video:* A video displaying MEA Viewer in action is available: <https://vimeo.com/143168058>

*Spike CSV Format:*

Spike data is stored in plain text comma-separated value (CSV) files which reside in the same directory as their associated HDF5 recordings and contain the same filename but with a .csv extension instead of .h5. Each file has a single header line detailing 5 columns: “electrode”, “time”, “amplitude”, “threshold”, “conductance”. Each row thereafter specifies a spike event where the electrode column contains the electrode ID, typically in the format A6.0 where A6 is the electrode identifier and 0 specifies the sorted spike group number on that electrode. A spike group number of -1 specifies spikes which could not be properly sorted. The time column specifies the time in seconds that the event occurred in the recording. The amplitude column specifies the amplitude of the event in uV. The threshold column specifies the spike detection threshold used for that channel in uV. The conductance column specifies *True* if this spike event is considered a redundant event due to it being part of a propagation signal, or *False* if it is not. A sample of the data format is given below:

electrode,time,amplitude,threshold,conductance

e12.0,48.861934661865234,-88.80604553222656,-36.17005157470703,False

e12.0,49.07347106933594,-74.42280578613281,-36.17005157470703,False

e12.0,49.107704162597656,-69.9498519897461,-36.17005157470703,True

e12.0,49.15875244140625,-74.18529510498047,-36.17005157470703,False

*Analog HDF5 Format*:

Analog data is stored in HDF5 files. For simplicity MEA Viewer expects data in the same format that is exported by the MultiChannel Systems DataManager conversion utility, though any data following the same structure is readily viewable. The HDF5 file should have an information table at the path /Data/Recording_0/AnalogStream/Stream_0/InfoChannel. This table should have at minimum the following columns:

InfoChannel Table

| **Column Name** | **Description** |
| --- | --- |
| Tick | Time period between samples in microseconds |
| ConversionFactor | The factor to multiply the raw data by to convert to Volts |
| Exponent | The exponent to apply to convert to Volts |
| Label | The electrode label |

The main analog data is stored in the /Data/Recording_0/AnalogStream/Stream_0/ChannelData path. This is a 2 dimensional table where each row corresponds to an electrode, and each column to successive data samples, taken at the sampling frequency specified by the Tick value in the InfoChannel table.

For a complete reference, sample data is provided (http://mea-tools.s3.amazonaws.com/MEAViewerSampleData.zip) and can be inspected using the free HDFView utility provided by the HDF Group (https://www.hdfgroup.org/downloads/hdfview/).
